# International study on inter-reader variability for circulating tumor cells in breast cancer

**DOI:** 10.1186/bcr3647

**Published:** 2014-04-23

**Authors:** Michail Ignatiadis, Sabine Riethdorf, François-Clement Bidard, Isabelle Vaucher, Mustapha Khazour, Françoise Rothé, Jessica Metallo, Ghizlane Rouas, Rachel E Payne, Raoul Charles Coombes, Ingrid Teufel, Ulrich Andergassen, Stella Apostolaki, Eleni Politaki, Dimitris Mavroudis, Silvia Bessi, Marta Pestrin, Angelo Di Leo, Michael Campion, Monica Reinholz, Edith Perez, Martine Piccart, Elin Borgen, Bjorn Naume, Jose Jimenez, Claudia Monica Aura, Laura Zorzino, Maria Cristina Cassatella, Maria Teresa Sandri, Bianca Mostert, Stefan Sleijfer, Jaco Kraan, Wolfgang Janni, Tanja Fehm, Brigitte Rack, Leon Terstappen, Madeline Repollet, Jean-Yves Pierga, Craig Miller, Christos Sotiriou, Stefan Michiels, Klaus Pantel

**Affiliations:** 1 Department of Medical Oncology, Institut Jules Bordet, Université Libre de Bruxelles, Brussels, Belgium; 2 Breast Cancer Translational Research Laboratory, Institut Jules Bordet, Université Libre de Bruxelles, Brussels, Belgium; 3 Institute of Tumor Biology, University Medical Center Hamburg-Eppendorf, Hamburg, Germany; 4 Department of Medical Oncology, Institut Curie & Université Paris Descartes, Paris, France; 5 Division of Cancer, Imperial College, Hammersmith Hospital, London, UK; 6 Department of Gynecology, University of Tubingen, Tubingen, Germany; 7 Department of Gynecology, Ludwig-Maximilians University of Munich, Munich, Germany; 8 Department of Medical Oncology, University of Crete, Heraklion, Greece; 9 Translational Research Unit, Hospital of Prato, Prato, Italy; 10 Department of Laboratory Medicine and Pathology, Mayo Clinic, Rochester, USA; 11 Department of Pathology, The Radium Hospital, Oslo, Norway; 12 Department of Oncology, Oslo University Hospital and K.G. Jebsen Centre for Breast Cancer Research, Institute for Clinical Medicine, University of Oslo, Oslo, Norway; 13 Molecular Pathology Lab, Vall d’Hebron Institut de Recerca (VHIR), Barcelona, Spain; 14 Division of Laboratory Medicine, European Institute of Oncology, Milan, Italy; 15 Department of Medical Oncology, Erasmus Medical Center, Rotterdam, The Netherlands; 16 Department of Gynecology, University of Ulm, Ulm, Germany; 17 Department of Gynecology, University of Düsseldorf, Düsseldorf, Germany; 18 Department of Medical Cell BioPhysics, Enschede, The Netherlands; 19 Veridex LCC, Philadelphia, USA; 20 Department of Biostatistics and Epidemiology, Institut Goustave Roussy, Paris, France

## Abstract

**Introduction:**

Circulating tumor cells (CTCs) have been studied in breast cancer with the CellSearch® system. Given the low CTC counts in non-metastatic breast cancer, it is important to evaluate the inter-reader agreement.

**Methods:**

CellSearch® images (N = 272) of either CTCs or white blood cells or artifacts from 109 non-metastatic (M0) and 22 metastatic (M1) breast cancer patients from reported studies were sent to 22 readers from 15 academic laboratories and 8 readers from two Veridex laboratories. Each image was scored as No CTC vs CTC HER2- vs CTC HER2+. The 8 Veridex readers were summarized to a Veridex Consensus (VC) to compare each academic reader using % agreement and kappa (κ) statistics. Agreement was compared according to disease stage and CTC counts using the Wilcoxon signed rank test.

**Results:**

For CTC definition (No CTC vs CTC), the median agreement between academic readers and VC was 92% (range 69 to 97%) with a median κ of 0.83 (range 0.37 to 0.93). Lower agreement was observed in images from M0 (median 91%, range 70 to 96%) compared to M1 (median 98%, range 64 to 100%) patients (*P* < 0.001) and from M0 and <3CTCs (median 87%, range 66 to 95%) compared to M0 and ≥3CTCs samples (median 95%, range 77 to 99%), (*P* < 0.001). For CTC HER2 expression (HER2- vs HER2+), the median agreement was 87% (range 51 to 95%) with a median κ of 0.74 (range 0.25 to 0.90).

**Conclusions:**

The inter-reader agreement for CTC definition was high. Reduced agreement was observed in M0 patients with low CTC counts. Continuous training and independent image review are required.

## Introduction

Circulating tumor cells (CTCs) can be identified in the peripheral blood of patients with solid tumors even in early disease stages and their detection and characterization have the potential to lead towards personalized treatment strategies in breast and other cancers [1-3].

Several technologies exist for CTC detection, but CellSearch® (Veridex, Raritan, NJ, USA) is the only one that has received Food and Drug Administration (FDA) clearance to be used as an aid in monitoring patients with metastatic breast, colorectal and prostate cancer. Compared to other CTC assays [4], CellSearch® is so far the only semi-automated system and has contributed considerably to the development of standards for CTC enumeration. In breast cancer, a multicenter study has shown that ≥5 CTCs/7.5 ml of blood were detected in 49% of 177 patients before starting a new treatment for metastatic disease and their detection was independently associated with worse progression-free and overall survival (PFS and OS) [5]. Subsequently, other studies have confirmed these results [6-8]. The value of CTC detection by CellSearch® in non-metastatic breast cancer has recently been emerging. Indeed, detection of ≥1 CTC/7.5 ml was reported in 23% of 115 patients with large operable and locally advanced breast cancer before neoadjuvant chemotherapy and was independently associated with worse outcome [9]. In another study, detection of ≥1 CTC/7.5 ml by CellSearch® at the time of surgery and before the administration of adjuvant chemotherapy in 24% of 302 patients was associated with decreased PFS and OS [10]. In the SUCCESS study, ≥1 CTC/23 ml were detected by CellSearch® in 21.5% of 2,026 patients with early breast cancer before adjuvant chemotherapy and the detection was independently associated with poor outcome [11]. In another study ≥1 CTC/30 ml of blood were detected in 19% of 404 patients with stage-I to -III breast cancer before surgery and their detection was independently associated with shorter distant disease-free survival [12]. Beyond enumeration, CTC characterization holds the promise to serve as a *liquid biopsy* to tailor treatment decisions [1,2]. As an example, as HER2 protein overexpression or gene amplification in the primary tumor is currently required for administering anti-HER2 treatment in breast cancer [13], HER2 status on CTCs has also been investigated in breast cancer using CellSearch® and other technologies [14-18].

More than 400 studies have included CTCs as a biomarker [19]. These include interventional studies assessing the value of CTCs as a treatment decision tool in the metastatic and non-metastatic setting [20,21]. Two studies have validated the analytical performance of CellSearch® for CTC detection in metastatic breast cancer [22,23]. In the metastatic setting, the main contributor to inter-laboratory variation was variability among the readers in image interpretation [23,24]. In non-metastatic breast cancer (BC) more than half of women with detectable CTCs have only 1 CTC/blood volume processed [9-12]. Therefore, in this setting, image interpretation is crucial, especially if CTC detection is to be used as a tool to decide on secondary adjuvant treatment strategies in the context of a clinical trial. Moreover, no study has addressed potential problems in image interpretation when HER2 expression on CTCs is investigated using CellSearch®, which is an important prerequisite for interventional studies using CTCs in trials testing HER2-directed therapies.

Therefore, we performed an international study to evaluate the inter-reader agreement in the classification of CellSearch® images as CTC and interpretation of HER2-staining on these images. Moreover, we came up with consensus guidelines for image interpretation in the non-metastatic breast cancer setting that were subsequently endorsed by CTC experts participating in this study.

## Methods

### Patient and samples

CellSearch® images from blood samples used for the detection of CTCs and characterization of CTC HER2 protein expression by CellSearch® from breast cancer patients were retrieved for this study. The blood samples were processed at one of the three different laboratories; the Institut Jules Bordet, Belgium (IJB), the Institut Curie, France (IC), and the University of Hamburg, Germany (UH), respectively. The IJB dataset included CellSearch® images from women with non-metastatic (M0) and metastatic (M1) breast cancer treated according to the standard of care in Belgium [17]. The IC dataset consisted of CellSearch® images from women with inflammatory M0 breast cancer participating in the BEVERLY-I and -II phase-2 trials [25,26]. The UH dataset included CellSearch® images from women receiving neoadjuvant chemotherapy in the GeparQuattro and GeparQuinto studies (M0 cohort) [16,27] and women from the Detect Study (M1 cohort) [18]. From each patient sample, a subset of CellSearch® images (images of either CTCs or white blood cells or artifacts) and not the full set of CellSearch® images were provided by investigators from the three independent, academic labs to the principal investigator at IJB who was responsible for the central coordination of the study. Due to technology limitations, at the time the study was initiated, it was not possible to send the full set of CellSearch® images from each patient sample. All CellSearch® images used in the present study are from previously reported studies of CTC detection in breast cancer [16-18,25-27]. All women participating in these studies provided informed consent and each study was approved by the respective institutions' ethical committees. The present study was approved by the IJB ethical committee (CE 2035). Informed consent for the present study was waived by the IJB ethical committee.

### Readers

The IJB lab sent the blinded CellSearch® images by email to twenty-two readers from one US and fourteen European academic laboratories as well as to eight readers from two Veridex laboratories. Each CellSearch® image was evaluated independently of the other images. All readers had already received the appropriate training provided by Veridex for the CellSearch® system, which includes training in image interpretation.

### Definition of CTC and HER2-positive CTC

In CellSearch®, a CTC is defined as an epithelial cell adhesion molecule (EpCAM)-positive cell (round to oval and sometimes polygonal or elongated in shape and at least 4 μm in size) that has positive immunofluoresence staining for a Cytokeratin (CK) (clone C11 and A53-B/A2) epithelial marker and positive staining for the nuclear dye 4',6-diamidino-2-phenylindole (DAPI) (the nuclear area should be smaller than the cytoplasmic area and at least 50% of the nucleus should be co-located with the cytoplasm). The CTC must also be negative for the leukocyte marker CD45. For this study, the readers were asked to score CTCs with HER2 immunofluoresence staining intensity of 2+, 3+ as HER2-positive and were provided with reference CellSearch® images from breast cancer cell lines (MCF7, BT20, T47D, MDA-MB-453, SKBR3, BT474) with different levels of HER2 expression according to Riethdorf *et al*. [16]. The readers scored each image as follows: No CTC, CTC/HER2-negative (CTC HER2-), or CTC/HER2-positive (CTC HER2+).

### Statistics

Results from the eight Veridex readers were summarized to a Veridex consensus (VC) in order to compare each academic reader using percentage (%) agreement and kappa (κ) statistics. We followed the Koch and Landis classification to interpret obtained κ values: concordance was considered bad if κ was less than 0.20, poor if κ was 0.20 to 0.40, moderate if κ was 0.40 to 0.60, good if κ was 0.60 to 0.80, and almost perfect if κ was above 0.81 [28]. VC was reached when there was >60% agreement between Veridex readers as to whether or not an image was a CTC. When there was <60% agreement by the Veridex readers for an image, this image was not included in any of the subsequent comparisons. If the VC was that the image was a CTC, then there had to be >60% agreement on the CTC being either HER2- or HER2+. If there was <60% agreement about the HER2 positivity or negativity among the Veridex readers for a CTC, this image was not included in any of the subsequent comparisons.

For each of the 22 academic readers, percentage (%) agreement with VC was calculated for the CTC detection question (no CTC versus CTC) and for the question of HER2 expression on CTCs (CTC HER2- versus CTC HER2+). In order to understand problems related to CellSearch® image interpretation, we compared the locations in the distributions of the agreement (%) between VC and the academic readers according to the dataset (IJB versus IC versus UH), disease stage (M0 versus M1), number of detected CTCs in the entire sample (≥5 CTCs versus <5 CTCs) and administration of systemic treatment before blood sampling (yes versus no) using non-parametric tests (Wilcoxon signed rank and Freedman test). Moreover, within M0 patient samples, we interrogated whether there was different agreement according to the number of CTCs detected in the entire sample (≥3 CTCs versus <3 CTCs) using the Wilcoxon signed rank test. Finally, we studied CellSearch® images with <70% agreement between VC and academic readers in order to understand reasons for discordance between readers.

## Results

### CellSearch® images and patient characteristics

A total of 272 CellSearch® images from 131 patients were analyzed. Table 1 provides information on relevant study demographics. The majority of CellSearch® images analyzed were from patients with M0 breast cancer, with <5 CTCs in their blood sample and from patients for whom blood sampling for CTC detection was performed before the administration of systemic treatment (Table 1).

**Table 1:** CellSearch® images and patient characteristics

	**Patients, number (%)**	**CellSearch® images, number (%)**
	**n = 131 (100%)**	**n = 272 (100%)**
**Dataset**		
Institute Jules Bordet	34 (26)	60 (22)
Institute Curie	60 (46)	114 (42)
University of Hamburg	37 (28)	98 (36)
**Age, years**		
≤50	58 (44)	108 (40)
>50	66 (51)	147 (54)
Unknown	7 (5)	17 (6)
**ER**		
Negative	54 (41)	106 (39)
Positive	68 (52)	139 (51)
Unknown	9 (7)	27 (10)
**HER2**		
Negative	103 (79)	193 (71)
Positive	15 (11)	44 (16)
Unknown	13 (10)	35 (13)
**Histology grade**		
1	5 (4)	6 (2)
2	46 (35)	85 (31)
3	69 (53)	147 (54)
Unknown	11 (8)	34 (13)
**Histology**		
Ductal	99 (76)	202 (74)
Lobular	10 (8)	12 (4)
Other	7 (5)	18 (7)
Unknown	15 (11)	40 (15)
**Stage**		
M0	109 (83)	215 (79)
M1	22 (17)	57 (21)
**CTC count**^**1**^		
<5 CTCs	90 (69)	150 (55)
≥5 CTCs	40 (30)	120 (44)
Unknown	2 (1)	2 (1)
**Systemic therapy before blood sample collection**^**2**^		
No	87 (65)	168 (62)
Yes	31 (24)	62 (23)
Unknown	15 (11)	42 (15)

^1^One patient with two samples (<5 CTCs and ≥ 5CTCs) was included; ^2^two patients with samples before and after treatment were included. ER, estrogen receptor; CTC, circulating tumor cell; M0, non-metastatic; M1, metastatic.

### Veridex consensus (VC)

For each image, the results from the eight Veridex readers were summarized to a VC, as explained in the Methods section. For CTC detection, VC was reached for 267 (98%) of 272 images with an average percent agreement for CTC versus no CTC between the eight Veridex readers of 97% (median 100%, range 63% to 100%). Of the 267 images, 157 (59%) were considered CTCs. For interpretation of HER2 staining on the CTC, VC was obtained for 155 (99%) out of 157 CTCs with an average percent agreement for HER2- versus HER2+ between the eight Veridex readers of 98% (median 100%, range 63% to 100%): 71 (46%) of 155 CTCs were considered as HER2 + .

### Agreement between academic readers and Veridex consensus for CTC definition

For the question of whether an image was a CTC (no CTC versus CTC), the median agreement between the academic readers and VC was 92% (range 69% to 97%), with a median κ statistic of 0.83 (range 0.37 to 0.93) (Table 2). We then asked whether agreement between the academic readers and VC was different according to the dataset (IJB versus IC versus UH), disease stage (M0 versus M1), CTC counts (<5 versus ≥5) and administration of systemic treatment before blood sampling (yes versus no). We observed significantly lower agreement between academic readers and VC for CellSearch® images from patients of the IJB dataset compared to the other datasets, from patients with M0 compared to M1 disease, <5 CTCs compared to ≥5 CTCs, and blood samples drawn after the administration of systemic treatment as compared to samples drawn before systemic treatment (Table 3, Additional file 1: Table S1 and Additional file 2: Table S2, online). CellSearch® images from samples with <5 CTCs were present more frequently in M0 (146 of 213 images, 68%) compared to M1 patients (4 of 57 images, 7%) (Pearson chi-square test, *P* <0.001) and in the IJB dataset, (52 of 60 images, 87%) compared to the UH (22 of 96 images, 22%) and the IC dataset (76 of 114 images, 67%) (*P* <0.001). When only images from M0 patients samples were analyzed, lower agreement was observed for images from samples with <3 CTCs (median 87%, range 66% to 95%) compared to those from samples where ≥ 3CTCs were detected (median 95%, range 77% to 99%), (*P* <0.001).

**Table 2:** Agreement (%) between academic readers and Veridex consensus (VC)

	**No CTC versus CTC**				**CTC HER2- versus CTC HER2+**			
**RDs**	**N**	**Agreement (%)**	**Kappa statistic**	**Kappa agreement**	**N**	**Agreement (%)**	**Kappa statistic**	**Kappa agreement**
A	267	91.8%	0.83	Almost perfect	155	81.3%	0.63	Substantial
B	267	91.4%	0.82	Almost perfect	155	81.3%	0.63	Substantial
C	267	92.5%	0.84	Almost perfect	155	90.3%	0.81	Almost perfect
D	267	91.8%	0.83	Almost perfect	155	71.6%	0.47	Moderate
E	266	95.1%	0.90	Almost perfect	154	93.5%	0.87	Almost perfect
F	267	92.1%	0.84	Almost perfect	155	87.1%	0.75	Substantial
G	267	91.4%	0.82	Almost perfect	155	91.0%	0.82	Almost perfect
H	267	91.4%	0.82	Almost perfect	155	88.4%	0.78	Substantial
I	267	92.1%	0.84	Almost perfect	155	91.6%	0.84	Almost perfect
J	267	83.9%	0.65	Substantial	155	76.8%	0.55	Moderate
K	220	87.3%	0.74	Substantial	117	79.5%	0.59	Moderate
L	266	92.9%	0.85	Almost perfect	154	94.8%	0.90	Almost perfect
M	220	87.3%	0.74	Substantial	117	90.6%	0.82	Almost perfect
N	267	95.9%	0.92	Almost perfect	155	92.3%	0.85	Almost perfect
O	267	96.6%	0.93	Almost perfect	155	92.9%	0.86	Almost perfect
P	267	94.4%	0.88	Almost perfect	155	85.2%	0.72	Substantial
Q	218	91.7%	0.83	Almost perfect	113	84.1%	0.69	Substantial
R	218	91.7%	0.83	Almost perfect	113	85.8%	0.73	Substantial
S	220	92.7%	0.85	Almost perfect	117	86.3%	0.73	Substantial
T	266	69.2%	0.37	Fair	154	51.3%	0.25	Fair
U	267	91.4%	0.82	Almost perfect	155	91.0%	0.82	Almost perfect
V	220	91.8%	0.84	Almost perfect	117	82.1%	0.66	Substantial

CTC, circulating tumor cell; RDs, readers; N, number of images. Kappa Agreement, below 0.00 Poor; 0.00 to 0.20 Slight; 0.21 to 0.40 Fair; 0.41 to 0.60 Moderate; 0.61 to 0.80 Substantial; 0.81 to 1.00 Almost Perfect.

**Table 3:** Percent agreement between academic readers and Veridex consensus (VC) overall and according to dataset, disease stage, circulating tumor cell (CTC) count and administration of systemic therapy before blood sample collection

	**Agreement, %**		
	**Median**	**Range**	***P*-value**
**All**	92	69 to 97	
**Dataset**			
Institute Jules Bordet	81	61 to 97	<0.001
Institute Curie	97	73 to 99	
University of Hamburg	94	69 to 98	
**Stage**			
M0	91	70 to 96	<0.001
M1	98	64 to 100	
**CTC count**			
<5 CTCs	88	66 to 95	<0.001
≥5 CTCs	97	74 to 100	
**Systemic therapy before blood sample collection**			
No	92	74 to 96	<0.001
Yes	89	55 to 97	

CTC, circulating tumor cell; M0, Non-metastatic, M1, metastatic.

### Agreement between academic readers and Veridex consensus (VC) for HER2 expression on CTCs

When only the images that were CTCs according to VC were analyzed for HER2 protein expression (CTC HER2- versus CTC HER2+), the median agreement between the academic readers and VC was 87% (range 51% to 95%), with a median κ statistic of 0.74 (range 0.25 to 0.90) (Table 2).

### Analysis of CellSearch® images with <70% agreement between academic readers and Veridex consensus (VC)

**Figure 1:**
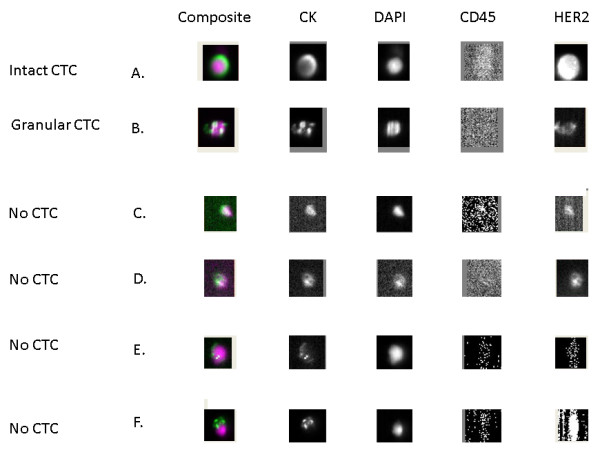
**Example of an intact circulating tumor cell (CTC) (A), a granular CTC (B) and of images with no CTCs (C,D,E,F) as they display the same morphology in all channels (C,D) or have a nucleus larger than the cytoplasm or not 50% inside the cytoplasm and cytokeratin staining not surrounding the nucleus (E,F).** DAPI, 4',6-diamidino-2-phenylindole.

There were many images with excellent agreement between academic readers and VC, such as images of intact CTCs (Figure 1A). In order to understand the reasons for discordance in image interpretation, we focused on images with <70% agreement between the academic readers and VC. We identified 25 images with <70% agreement for the CTC question (CTC versus no CTC) and 14 images with <70% agreement for the HER2 expression question (HER2- versus HER2+ CTCs). Disagreement was observed for images of CTCs with CK staining that surrounded the nucleus but was either incomplete or granular (Figure 1B). A total of 16 of the 25 images with <70% agreement for the CTC question were considered by some academic readers as CTCs, whereas the VC was that these images were not CTCs. These included images displaying the same morphology in all channels (Figure 1C,D) or a nucleus larger than the cytoplasm or <50% inside the cytoplasm and with cytokeratin (CK) staining not surrounding the nucleus (Figure 1E,F). Images with <70% agreement between academic readers and VC for the HER2 expression question were images from CTCs with intermediate HER2 staining (neither too strong nor absent).

## Discussion

To our knowledge, this is the first study to evaluate the inter-reader agreement for CTC detection in both M1 and M0 breast cancer in a large international multicenter study comprising 15 centers from Europe and the United States. Our results show that the inter-reader agreement is high for CTC definition and for characterizing HER2 expression on CTCs.

Lower inter-reader agreement for CTC definition was observed for CellSearch® images of samples from patients with M0 disease, with low CTC counts, samples from the IJB dataset, and samples drawn after the administration of systemic treatment. CellSearch® images of samples with low CTC counts were more frequently observed in the IJB compared to the other datasets and in M0 compared to M1 disease and this can partly explain the lower inter-reader agreement in these groups. In a recently reported study of CTC detection by CellSearch® at the time of surgery in patients with early breast cancer, increasing CTC counts were associated with increasing hazard ratio (HR) for disease progression. The presence of ≥1 CTC, ≥2 CTCs and ≥3 CTCs was associated with a HR of 4.6, 5.5 and 6.7, respectively [10]. Although, this increase in HRs can be attributed to the increase in the number of CTCs, the data from the present analysis imply that this might be also due to the lower false CTC-positive cases in women with increasing CTC counts. Finally, a possible explanation for the lower inter-reader agreement in samples drawn after the administration of systemic treatment is the presence in these samples of granular CTCs that have been demonstrated to be mainly apoptotic, and to be a source of inter-reader disagreement in a previous study [23].

There are some limitations in the way the present validation study was conducted. Although CellSearch® image selection was performed by three independent, academic laboratories, a selection bias towards either CellSearch® images that are easy (typical CTC images) or difficult to interpret (not typical CTC images) cannot be excluded. Moreover, the readers were provided with a subset of CellSearch® images (images of either CTCs or white blood cells or artifacts) and not the full set of CellSearch® images from each patient sample. Therefore, a bias in image selection cannot be excluded. Moreover, the readers were scoring images but were not able to use the Cell Select tool of the CellSearch® system, which allows relocation of a cellular image in the cartridge and study of its characteristics in more detail. As the technology evolves, one can imagine that the exchange of CellSearch® images for central image review will be more easily performed.

The above limitations may partly explain some of the discrepancies observed between readers. Although overall inter-reader agreement was high, agreement was lower in images from M0 patients with <3 CTCs. In order to improve the performance of academic readers, especially in the setting of non-metastatic breast cancer with low CTC counts, we propose the following: 1) images displaying identical morphology in all channels (CK, DAPI and HER2 if performed) or images with a nucleus larger than the cytoplasm or <50% inside the cytoplasm should not be considered as CTCs; 2) the authors reporting studies on CTCs using CellSearch® should clearly indicate whether their CTC definition includes only intact CTCs (complete CK staining surrounding the nucleus) or whether it also includes granular CTCs (CK staining that surrounds the nucleus but is either granular or incomplete); 3) in cases of blood samples from M0 patients with low CTC counts (<3 CTCs) evaluation of images by at least two independent readers - ideally from different labs - should be performed. Such an independent image review is applied in the ongoing European Organization for Research and Treatment of Cancer (EORTC) Treat CTC trial (ClinicalTrials.gov Identifier: NCT01548677). This trial is testing the value of CTC detection to decide for secondary adjuvant treatment in BC.

We propose that investigators conducting studies on CTCs in non-metastatic breast cancer follow these guidelines for CellSearch® image interpretation. The present study might also be a model for similar experiments regarding other image-based CTC technologies. This could facilitate consistent result interpretation across studies on CTC detection and characterization, and accelerate the clinical development of CTC-related biomarkers.

## Conclusions

This is the first study to evaluate the inter-reader agreement for CTC detection in both metastatic and non-metastatic BC in a large international multicenter study comprising 15 centers from Europe and the United States. We demonstrated that inter-reader agreement using CellSearch® was high overall but was reduced in non-metastatic breast cancer patients with low CTC counts. This study resulted in consensus guidelines for image interpretation for CTC detection in non-metastatic breast cancer. Continuous training, independent image review and adherence to these consensus guidelines should be considered in studies evaluating the clinical utility of CTC-related biomarkers in non-metastatic breast cancer.

## Abbreviations

BC: breast cancer; CK: cytokeratin; CTCs: circulating tumor cells; DAPI: 4',6-diamidino-2-phenylindole; EORTC: European Organization for Research and Treatment of Cancer; EpCAM: epithelial cell adhesion molecule; FDA: Food and Drug Administration; HR: hazard ratio; IC: Institut Curie; IJB: Institut Jules Bordet; M0: non-metastatic; M1: metastatic; OS: overall survival; PFS: progression-free survival; UH: University of Hamburg; VC: Veridex consensus.

## Competing interests

Michail Ignatiadis, Francois-Clement Bidard, Stefan Sleijfer, Wolfgang Janni, Brigitte Rack, Leon Terstappen, Jean-Yves Pierga and Klaus Pantel have received research support from CellSearch®, Veridex. Michail Ignatiadis, Francois-Clement Bidard, Stefan Sleijfer, Leon Terstappen, Jean-Yves Pierga and Klaus Pantel have received consultancy fees from CellSearch®, Veridex. All other authors have no conflict of interest to declare.

## Authors’ contributions

MI, CM, SM, KP were involved in study conception and design. All authors were involved in acquisition, analysis, or interpretation of data for this study. MI wrote the manuscript. All authors provided input by revising the manuscript critically for important intellectual content. All authors read and approved the final version of the manuscript.

## Supplementary Material

Additional file 1: Table S1 Agreement (%) between academic readers and Veridex consensus (VC) according to dataset and disease stage. Description of data: we observed significantly lower agreement between academic readers and VC for CellSearch® images from patients of the Institut Jules Bordet (IJB) dataset compared to the other datasets and from patients with non-metastatic (M0) compared to metastatic (M1) disease. Click here for file

Additional file 2: Table S2 Agreement (%) between academic readers and Veridex consensus (VC) according to circulating tumor cell (CTC) count and administration of systemic therapy before blood sample collection. Description of data: we observed significantly lower agreement between academic readers and VC for CellSearch® images from patients with <5 CTCs compared to ≥5 CTCs and blood samples drawn after the administration of systemic treatment as compared to samples drawn before systemic treatment. Click here for file
