# Supplementary material for: International study on inter-reader variability for circulating tumor cells in breast cancer
Source: Breast Cancer Res. 2014 Apr 23;16(2):R43. doi: 10.1186/bcr3647 (PMC4052944; doi:10.1186/bcr3647)
Supplement: Additional file 1: Table S1 — Agreement (%) between academic readers and Veridex consensus (VC) according to dataset and disease stage. Description of data: we observed significantly lower agreement between academic readers and VC for CellSearch® images from patients of the Institut Jules Bordet (IJB) dataset compared to the other datasets and from patients with non-metastatic (M0) compared to metastatic (M1) disease. [file bcr3647-S1.docx]

| **Additional Table 1. Agreement (%) between academic readers and Veridex Consensus (VC) according to dataset and disease stage** | | | | | | |
| --- | --- | --- | --- | --- | --- | --- |
|  | |  |  |  |  |  |
|  |  |  |  |  |  |  |
|  | **Dataset** | | | | | |
|  | **Institut Jules Bordet (IJB)** | | **Institut Curie (IC)** | | **University of Hamburg (UH)** | |
| **Academic Readers** | **No of Images** | **Agreement (%)** | **No of Images** | **Agreement (%)** | **No of Images** | **Agreement (%)** |
| **A** | 59 | 81.4% | 112 | 97.3% | 96 | 91.7% |
| **B** | 59 | 78.0% | 112 | 97.3% | 96 | 92.7% |
| **C** | 59 | 76.3% | 112 | 98.2% | 96 | 95.8% |
| **D** | 59 | 88.1% | 112 | 96.4% | 96 | 88.5% |
| **E** | 59 | 89.8% | 111 | 97.3% | 96 | 95.8% |
| **F** | 59 | 78.0% | 112 | 96.4% | 96 | 95.8% |
| **G** | 59 | 76.3% | 112 | 97.3% | 96 | 93.8% |
| **H** | 59 | 76.3% | 112 | 97.3% | 96 | 93.8% |
| **I** | 59 | 83.1% | 112 | 94.6% | 96 | 94.8% |
| **J** | 59 | 61.0% | 112 | 90.2% | 96 | 90.6% |
| **K** | 59 | 69.5% | 112 | 92.0% | 49 | 98.0% |
| **L** | 59 | 86.4% | 111 | 96.4% | 96 | 92.7% |
| **M** | 59 | 74.6% | 112 | 92.9% | 49 | 89.8% |
| **N** | 59 | 94.9% | 112 | 97.3% | 96 | 94.8% |
| **O** | 59 | 96.6% | 112 | 97.3% | 96 | 95.8% |
| **P** | 59 | 93.2% | 112 | 94.6% | 96 | 94.8% |
| **Q** | 59 | 81.4% | 112 | 99.1% | 47 | 87.2% |
| **R** | 59 | 81.4% | 112 | 99.1% | 47 | 87.2% |
| **S** | 59 | 83.1% | 112 | 95.5% | 49 | 98.0% |
| **T** | 59 | 62.7% | 111 | 73.0% | 96 | 68.8% |
| **U** | 59 | 76.3% | 112 | 97.3% | 96 | 93.8% |
| **V** | 59 | 84.7% | 112 | 93.8% | 49 | 95.9% |
|  |  |  |  |  |  |  |
|  |  |  |  |  |  |  |
|  |  |  |  |  |  |  |
|  |  |  |  |  |  |  |
|  | **Disease stage** | | | |  |  |
|  | **Non-metastatic (M0)** | | **Metastatic (M1)** | |  |  |
| **Academic Readers** | **No of Images** | **Agreement (%)** | **No of Images** | **Agreement (%)** |  |  |
| **A** | 211 | 89.6% | 56 | 100.0% |  |  |
| **B** | 211 | 89.1% | 56 | 100.0% |  |  |
| **C** | 211 | 90.5% | 56 | 100.0% |  |  |
| **D** | 211 | 90.5% | 56 | 96.4% |  |  |
| **E** | 210 | 94.3% | 56 | 98.2% |  |  |
| **F** | 211 | 90.5% | 56 | 98.2% |  |  |
| **G** | 211 | 89.1% | 56 | 100.0% |  |  |
| **H** | 211 | 89.6% | 56 | 98.2% |  |  |
| **I** | 211 | 90.5% | 56 | 98.2% |  |  |
| **J** | 211 | 80.6% | 56 | 96.4% |  |  |
| **K** | 164 | 83.5% | 56 | 98.2% |  |  |
| **L** | 210 | 91.9% | 56 | 96.4% |  |  |
| **M** | 164 | 86.0% | 56 | 91.1% |  |  |
| **N** | 211 | 95.3% | 56 | 98.2% |  |  |
| **O** | 211 | 96.2% | 56 | 98.2% |  |  |
| **P** | 211 | 92.9% | 56 | 100.0% |  |  |
| **Q** | 208 | 91.3% | 10 | 100.0% |  |  |
| **R** | 208 | 91.3% | 10 | 100.0% |  |  |
| **S** | 164 | 90.9% | 56 | 98.2% |  |  |
| **T** | 210 | 70.5% | 56 | 64.3% |  |  |
| **U** | 211 | 89.1% | 56 | 100.0% |  |  |
| **V** | 164 | 90.2% | 56 | 96.4% |  |  |
|  |  |  |  |  |  |  |
