# Supplementary material for: International study on inter-reader variability for circulating tumor cells in breast cancer
Source: Breast Cancer Res. 2014 Apr 23;16(2):R43. doi: 10.1186/bcr3647 (PMC4052944; doi:10.1186/bcr3647)
Supplement: Additional file 2: Table S2 — Agreement (%) between academic readers and Veridex consensus (VC) according to circulating tumor cell (CTC) count and administration of systemic therapy before blood sample collection. Description of data: we observed significantly lower agreement between academic readers and VC for CellSearch® images from patients with <5 CTCs compared to ≥5 CTCs and blood samples drawn after the administration of systemic treatment as compared to samples drawn before systemic treatment. [file bcr3647-S2.docx]

| **Additional Table 2. Agreement (%) between academic readers and Veridex Consensus (VC) according to** | | | | | | | | |  |  |  |
| --- | --- | --- | --- | --- | --- | --- | --- | --- | --- | --- | --- |
| **Circulating Tumor Cell (CTC) count and administration of systemic therapy before blood sample collection** | | | | | | | |  |  |  |  |
|  |  |  |  |  |  |  |  | |  |  |  |
|  |  |  |  |  |  |  |  | |  |  |  |
|  | **CTC count** | | | |  |  | **Systemic therapy before blood sample collection** | | | | |
|  | **<5** | | **≥5** | |  |  | **No** | | | **Yes** | |
| **Academic Readers** | **No of Images** | **Agreement (%)** | **No of Images** | **Agreement (%)** |  | **Academic Readers** | **No of Images** | | **Agreement (%)** | **No of Images** | **Agreement (%)** |
| **A** | 147 | 88.4% | 118 | 95.8% |  | **A** | 165 | | 90.9% | 60 | 90.0% |
| **B** | 147 | 87.1% | 118 | 96.6% |  | **B** | 165 | | 89.7% | 60 | 91.7% |
| **C** | 147 | 86.4% | 118 | 100.0% |  | **C** | 165 | | 92.7% | 60 | 90.0% |
| **D** | 147 | 89.8% | 118 | 94.9% |  | **D** | 165 | | 93.9% | 60 | 88.3% |
| **E** | 147 | 91.8% | 117 | 99.1% |  | **E** | 164 | | 96.3% | 60 | 90.0% |
| **F** | 147 | 87.8% | 118 | 97.5% |  | **F** | 165 | | 92.1% | 60 | 88.3% |
| **G** | 147 | 85.7% | 118 | 98.3% |  | **G** | 165 | | 91.5% | 60 | 90.0% |
| **H** | 147 | 88.4% | 118 | 95.8% |  | **H** | 165 | | 91.5% | 60 | 88.3% |
| **I** | 147 | 87.1% | 118 | 98.3% |  | **I** | 165 | | 92.1% | 60 | 88.3% |
| **J** | 147 | 72.1% | 118 | 98.3% |  | **J** | 165 | | 84.2% | 60 | 78.3% |
| **K** | 128 | 78.9% | 92 | 98.9% |  | **K** | 139 | | 87.1% | 54 | 81.5% |
| **L** | 147 | 89.1% | 117 | 97.4% |  | **L** | 164 | | 95.1% | 60 | 86.7% |
| **M** | 128 | 82.8% | 92 | 93.5% |  | **M** | 139 | | 88.5% | 54 | 79.6% |
| **N** | 147 | 93.9% | 118 | 98.3% |  | **N** | 165 | | 95.8% | 60 | 95.0% |
| **O** | 147 | 95.2% | 118 | 98.3% |  | **O** | 165 | | 96.4% | 60 | 96.7% |
| **P** | 147 | 93.2% | 118 | 95.8% |  | **P** | 165 | | 94.5% | 60 | 93.3% |
| **Q** | 144 | 90.3% | 72 | 95.8% |  | **Q** | 162 | | 93.8% | 35 | 88.6% |
| **R** | 144 | 90.3% | 72 | 95.8% |  | **R** | 162 | | 93.8% | 35 | 88.6% |
| **S** | 128 | 88.3% | 92 | 98.9% |  | **S** | 139 | | 92.1% | 54 | 90.7% |
| **T** | 147 | 66.0% | 117 | 73.5% |  | **T** | 164 | | 73.8% | 60 | 55.0% |
| **U** | 147 | 85.7% | 118 | 98.3% |  | **U** | 165 | | 91.5% | 60 | 90.0% |
| **V** | 128 | 89.8% | 92 | 94.6% |  | **V** | 139 | | 92.1% | 54 | 88.9% |
